# Exploring the Use of Pseudosymmetry in the Design of Higher-Symmetry Crystals of Racemic Compounds

**DOI:** 10.1021/acs.cgd.4c01240

**Published:** 2024-11-25

**Authors:** Brent Lindquist-Kleissler, Viky Villanueva, Addis Getahun, Timothy C. Johnstone

**Affiliations:** Department of Chemistry and Biochemistry, University of California Santa Cruz, Santa Cruz, California 95064, United States

## Abstract

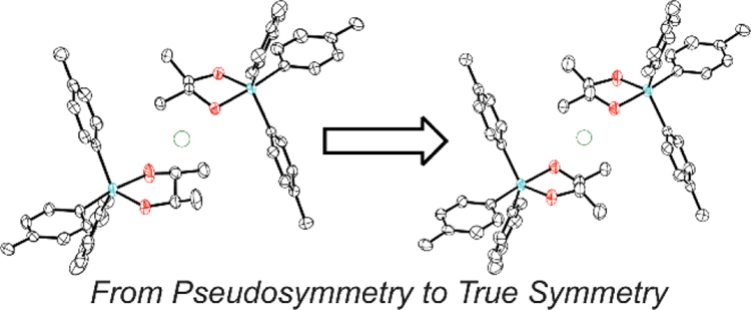

Organometallic antimony(V) complexes were prepared as model compounds to better understand the interactions of chiral chelating diols with this metalloid. These complexes feature three aryl groups (*meta*-xylyl or *para*-tolyl) and a bidentate *trans*-2,3-butanediolate. The *meta*-xylyl and *para*-tolyl complexes of either enantiomerically pure 2*R*,3*R*-butanediolate or 2*S*,3*S*-butanediolate (compounds **1**–**4**) crystallized in Sohncke space groups, as expected. In each case, though, pseudoinversion centers were present that mimic higher-symmetry space groups through global pseudosymmetry. We hypothesized that the crystallization of 1:1 mixtures of the enantiomeric complexes would produce crystals in the centrosymmetric space group approximated by the pseudosymmetry. The enantiomerically pure *meta*-xylyl complexes each crystallized in space group *P*1 (approximating *P*1̅), and the racemic compound did indeed crystallize in *P*1̅. The enantiomerically pure *para*-tolyl complexes each crystallized in space group *P*2_1_ (approximating *P*2_1_/*c*), but the racemic compound crystallized in *P*1̅. Although the enantiomerically pure and racemic compounds are not isostructural, there are similarities in their 3D structures that are analyzed.

## Abstract 2

A panel of organometallic antimony(V) compounds is explored that bear chiral chelating diolate ligands. The enantiomerically pure compounds crystallize with pseudoinversion centers in a manner that mimics higher-symmetry space groups. We investigated whether the racemic compound would crystallize in this higher-symmetry space group.

## Introduction

It is a curiously interesting feature of molecular crystals that in most cases *Z*′ = 1. That is, the entire crystal can be generated by the application of the symmetry operations of the space group on a single molecule in a single conformation. This predisposition for molecules to crystallize with *Z*′ = 1 has been recognized since the earliest days of molecular crystallography. One striking example, which begins before the discovery of X-rays, concerns Adolf von Baeyer’s 1886 description of a crystalline product formed from the condensation of pyrrole and acetone, which was originally formulated as the dimeric C_14_H_18_N_2_.^1^ Classical morphological measurements on these crystals in 1888 indicated that they were tetragonal,^2^ and this crystal system was later confirmed when Laue and spectral photographs were used to calculate accurate unit cell parameters.^3^ Given the unit cell dimensions and symmetry of the Laue photographs, dimeric C_14_H_18_N_2_ would have crystallized with *Z*′ = 2. Even at the time, this situation was sufficiently unexpected that an ebullioscopic molecular weight determination was conducted, which revealed that the molecular product was, in fact, tetrameric C_24_H_36_N_4_.^3^ A much later solution and refinement of the structure confirmed that the porphyrinogen product was a tetrameric calix[4]pyrrole that crystallized with *Z*′ = 1.^4^

Despite the predominance of *Z*′ = 1 structures, there are many instances in which the asymmetric unit of a molecular crystal contains more than one formula unit. A 2008 survey of the small-molecule structures deposited in the Cambridge Structural Database revealed that *Z*′ ≥ 2 for 12.2% of entries.^5^ In approximately one-fifth of these cases, the two molecules differ substantially (>5%) in one of their moments of inertia, which is a computationally efficient means of rapidly assessing whether molecules are in different configurations and therefore cannot be related to each other by symmetry. It was estimated that, in the remaining structures, over 80% exhibited some approximate (<0.5 Å atom^–1^) relationship between the molecules.

**Figure 1:**
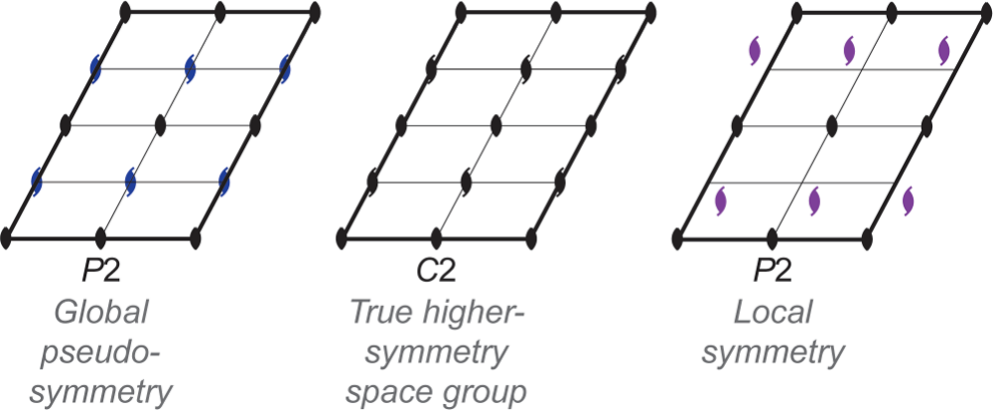
Left: A crystal with an arrangement of 2-fold rotations (black) corresponding to space group *P*2 and with pseudo-2_1_ screw axes (blue). The blue symmetry elements are not truly present; the asymmetric unit of the *P*2 structure could, for instance, contain two molecules in slightly different conformations that are approximately related to one another by a 2_1_ screw. The position of the pseudo-2_1_ screw axes is such that, if they were rigorously obeyed, the structure would belong to the higher-symmetry space group *C*2 (center). Right: A crystal with an arrangement of 2-fold rotations (black) corresponding to space group *P*2 and with local 2_1_ screw axes (purple). The purple symmetries apply only locally and not to the entirety of the crystal. The local symmetry relationships (purple) may approximately relate molecules that are in different conformations (as with the blue symmetries at left) or may be exact within experimental error. In either case, they do not coincide with the locations of symmetries from a higher-symmetry space group.

A useful classification scheme divides these cases into two categories: global pseudosymmetry and local symmetry.^6^ Consider a crystal that belongs to some space group *G* and features molecules that are almost, but not quite, related by a symmetry operation that is *not* an element of the space group *G*. Global pseudosymmetry describes those situations where, if the additional pseudosymmetry was strictly obeyed, its addition to space group *G* would afford higher-symmetry space group *H* (Figure 1). Local symmetry describes a relationship that maps one portion of the asymmetric unit onto another, but not onto the rest of the crystal.^7^ The local symmetry mapping can be approximate or exact (within experimental error). In contrast to global pseudosymmetry, however, even if the additional symmetry is strictly obeyed, its addition to space group *G* does not afford a valid higher-symmetry space group (Figure 1).

Investigation of pseudosymmetry can have important practical ramifications and undiagnosed global pseudosymmetry can lead to complications during refinement,^8,9^ misassigned stereochemistry in pharmaceutically active agents,^10^ entrapment in false refinement minima,^11^ and a host of other crystallographic pathologies.^12,13^ Pseudosymmetry has also been investigated because it provides fundamental insight into how and why molecules crystallize in the ways that they do.^14^ An analysis of database-deposited small-molecule crystal structures revealed that the incidence of *Z*′ > 1 structures decreases with increasing *Z*′, but that there are spikes when *Z*′ is even.^15^ A potential implication of this observation is that the molecules in the asymmetric unit are preferentially related to each other by pairwise symmetries (e.g., inversion, 2_1_ screw, glide, etc.).^16^

We recently reported an investigation of organometallic Sb(V) complexes that serve as models of the species proposed to be the active agents in the antileishmanial pentavalent antimonial drugs.^17^ This series of compounds comprised triarylantimony(V) fragments bound to vicinal diolates of varying stereochemistry. During the characterization of Sb(*p*-Tol)_3_L where L = 2*R*,3*R*-butanediolate, 1*S*,2*S*-cyclohexanediolate, or *S*-1,2-propanediolate, we noted that all three crystal structures featured two molecules in the asymmetric unit and that in every case the crystallographically independent molecules were related by a pseudoinversion center. Furthermore, all three were instances of global pseudosymmetry; the pseudoinversion centers held the positions of true crystallographic inversion centers in higher-symmetry space groups.

**Chart 1:**
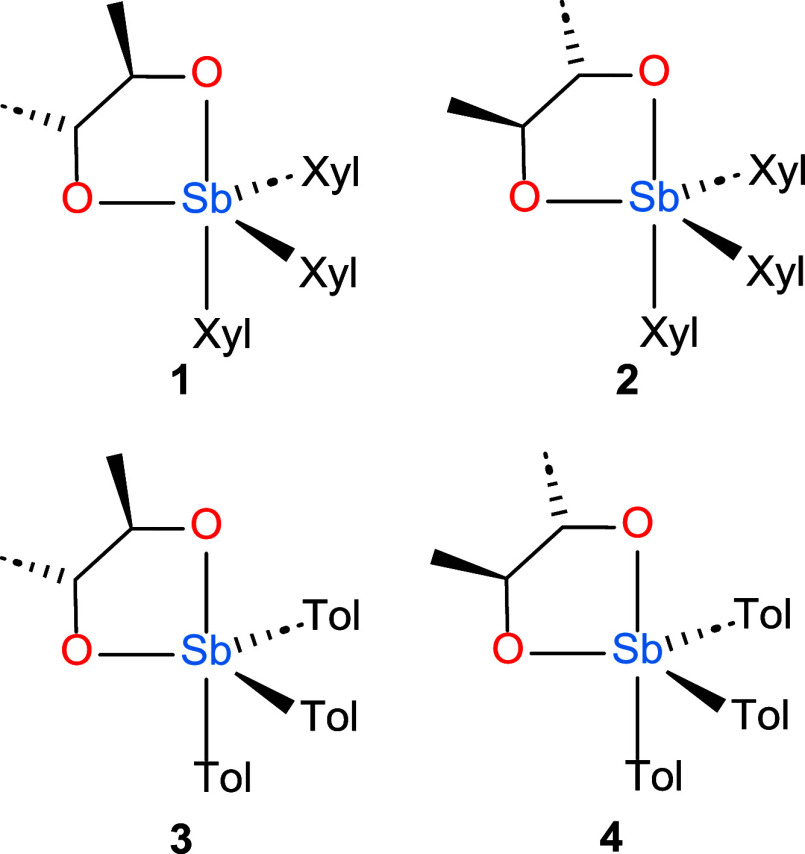
Enantiomerically Pure Compounds Investigated in the Present Work

In this present work, we sought to test the hypothesis that growing crystals of such substances from a racemic mixture would produce crystals of the racemic compound in which the overall crystal structure of the enantiomerically pure species was preserved but in which the pseudoinversion centers are converted to true crystallographic inversion centers. Consequently, the space group of the racemic compound would be the supergroup approximated by the crystals of the enantiomerically pure compound. Specifically, we explored complexes of *trans*-2,3-butanediolates with two different triarylantimony(V) scaffolds, where aryl = *meta*-xylyl or *para*-tolyl (Chart 1).

## Experimental Section

### General Methods

All solvents and reagents were commercially available and used as received, unless stated otherwise. Sb(*p*-Tol)_3_, Sb(*m*-Xyl)_3_, and Sb(*p*-Tol)_3_Br_2_ were prepared as previously described.^18,19^ Et_2_O was dried by using 3-Å molecular sieves. CDCl_3_ was purchased from Cambridge Isotope Laboratories and was used as received. ^1^H and ^13^C{^1^H} NMR spectra were recorded on a Bruker Avance III HD 500 MHz NMR spectrometer equipped with a multinuclear Smart Probe. Chemical shifts in the ^1^H and ^13^C{^1^H} NMR spectra are reported in ppm as chemical shifts from tetramethylsilane and were referenced using the CHCl_3_ (^1^H, 7.26 ppm) and CDCl_3_ (^13^C, 77.0 ppm) solvent signals. *J* values are reported as absolute values in Hz. Elemental analyses were performed by Micro-Analysis, Inc. (Wilmington, DE) and Midwest Microlabs (Indianapolis, IN).

### Synthesis of Sb(*m*-Xyl)_3_Br_2_

Sb(*m*-Xyl)_3_ (982 mg, 2.25 mmol) was dissolved in DCM (20 mL). A solution of Br_2_ (430 mg, 2.70 mmol) in DCM (2 mL) was added in a dropwise manner to the stibine solution. The reaction mixture was stirred at room temperature for 1 h, layered with 80 mL of hexanes, and allowed to stand at −20 °C overnight. The colorless crystals that formed were washed with cold hexanes and dried in air, yield: 826 mg, 62%. ^1^H NMR (500 MHz, CDCl_3_, δ) 7.72 (s, 6H, *o*-CH), 7.15 (s, 3H, *p*-H), 2.40 (s, 18H, *m*-CH_3_) ppm. ^13^C{^1^H} NMR (126 MHz, CDCl_3_, δ): 140.8, 139.4, 133.5, 21.7 ppm.

### General Synthesis of **1**–**4**

The triaryldihalostiborane (0.54 mmol), Sb(*m*-Xyl)_3_Br_2_ for **1** and **2** and Sb(*p*-Tol)_3_Br_2_ for **3** and **4**, was dissolved in DCM (5 mL). A solution of the appropriate 2,3-butanediol (0.54 mmol) in DCM (2 mL) was added, followed by Et_3_N (1.08 mmol). The reaction mixture was stirred at room temperature for 1 h and then stripped of solvent under reduced pressure to yield a white solid. The product was extracted from the solid with Et_2_O (3 × 2 mL), and the solvent was removed under reduced pressure to yield a colorless oil. The oil was dissolved in MeCN (20 mL) and allowed to stand at −20 °C for 12 h. The product was collected as colorless crystals on a Hirsch funnel, washed with cold MeCN, and dried by passing air through the filter cake for 15 min. Yields and characterization data are provided below.

#### Compound **1**

Prepared with Sb(*m*-Xyl)_3_Br_2_ and 2*R*,3*R*-butanediol. Yield 132 mg, 50%. ^1^H NMR (500 MHz, CDCl_3_, δ) 7.26 (s, 6H, Ar-H), 7.04 (s, 3H, Ar-H), 3.40 (m, 2H, CH), 2.28 (s, 18H, CH_3_), 1.22 (d, *J* = 5.6, 6H, CH_3_) ppm. ^13^C{^1^H} NMR (126 MHz, CDCl_3_, δ): 139.3, 138.2, 132.7, 132.2, 72.2, 21.6, 21.2 ppm. Elemental analysis calcd (%) for SbC_28_H_35_O_2_: C 64.02, H 6.72; found: C 64.07, H 6.73.

#### Compound **2**

Prepared with Sb(*m*-Xyl)_3_Br_2_ and 2*S*,3*S*-butanediol. Yield 147 mg, 56%. ^1^H NMR (500 MHz, CDCl_3_, δ) 7.26 (s, 6H, Ar-H), 7.04 (s, 3H, Ar-H), 3.40 (m, 2H, CH), 2.28 (s, 18H, CH_3_), 1.22 (d, *J* = 5.6, 6H, CH_3_) ppm. ^13^C{^1^H} NMR (126 MHz, CDCl_3_, δ): 139.3, 138.2, 132.7, 132.2, 72.1, 21.6, 21.2 ppm. Elemental analysis calcd (%) for SbC_28_H_35_O_2_: C 64.02, H 6.72; found: C 64.08, H 6.71.

#### Compound **3**

Prepared with Sb(*p*-Tol)_3_Br_2_ and 2*R*,3*R*-butanediol. Yield 191 mg, 73%. NMR spectroscopic data match those previously reported.^17^^1^H NMR (500 MHz, CDCl_3_, δ) 7.55 (d, *J* = 8.0, 6H, Ar-H), 7.20 (d, ^3^*J* = 7.6, 6H, Ar-H), 3.38 (m, 2H, CH), 2.36 (s, 9H, CH_3_), 1.20 (d, *J* = 5.6, 6H, CH_3_) ppm. ^13^C{^1^H} NMR (126 MHz, CDCl_3_, δ): 140.5, 136.1, 135.2, 129.7, 72.1, 21.6, 21.1 ppm. Elemental analysis calcd (%) for SbC_25_H_29_O_2_: C 62.13, H 6.05; found: C 62.00, H 6.21.

#### Compound **4**

Prepared with Sb(*p*-Tol)_3_Br_2_ and 2*S*,3*S*-butanediol. Yield 155 mg, 60%. ^1^H NMR (500 MHz, CDCl_3_, δ) 7.55 (d, *J* = 8.0, 6H, Ar-H), 7.20 (d, *J* = 7.7, 6H; Ar-H), 3.38 (m, 2H, CH), 2.36 (s, 9H, CH_3_), 1.20 (d, *J* = 5.6, 6H, CH_3_) ppm. ^13^C{^1^H} NMR (126 MHz, CDCl_3_, δ) 140.5, 136.1, 135.2, 129.7, 72.1, 21.6, 21.1 ppm. Elemental analysis calcd (%) for SbC_25_H_29_O_2_: C 62.13, H 6.05; found: 62.23, H 6.06.

### X-Ray Crystallography

Crystals of Sb(*m*-Xyl)_3_ and Sb(*m*-Xyl)_3_Br_2_ were grown from DCM/hexanes at room temperature. Crystals of **1**–**4** were grown by cooling an MeCN solution of the compound to −20 °C overnight. Crystals of racemic compounds **1**/**2** and **3**/**4** were grown by cooling MeCN solutions that contained equal amounts (3 mM) of each enantiomer to −20 °C overnight. X-ray diffraction-quality crystals of each were selected under a microscope, loaded onto a MiTeGen polyimide loop using paratone-*n*, and mounted onto a Rigaku XtaLAB Synergy-S single crystal diffractometer. Each crystal was cooled to 100 K under a stream of nitrogen. Diffraction of Cu Kα radiation from a PhotonJet-S microfocus source was detected by using a HyPix-6000HE hybrid photon counting detector. Screening, indexing, data collection, and data processing were performed with CrysAlisPro. The structures were solved using SHELXT and refined using SHELXL according to established procedures.^20−22^ All non-H atoms were refined anisotropically. H atoms were placed at geometrically calculated positions and refined with a riding model. The U_iso_ values of the H atoms were set equal to 1.2(*U*_eq_) of the C atoms to which they are attached for CH_2_ and aromatic CH units, or 1.5(*U*_eq_) for methyl groups. Fingerprint diagrams were prepared using CrystalExplorer.^23−26^

## Results

### Synthesis and Characterization of (2*R*,3*R*-Butanediolato)tris(*meta*-xylyl)stiborane (**1**)

**Scheme 1:**
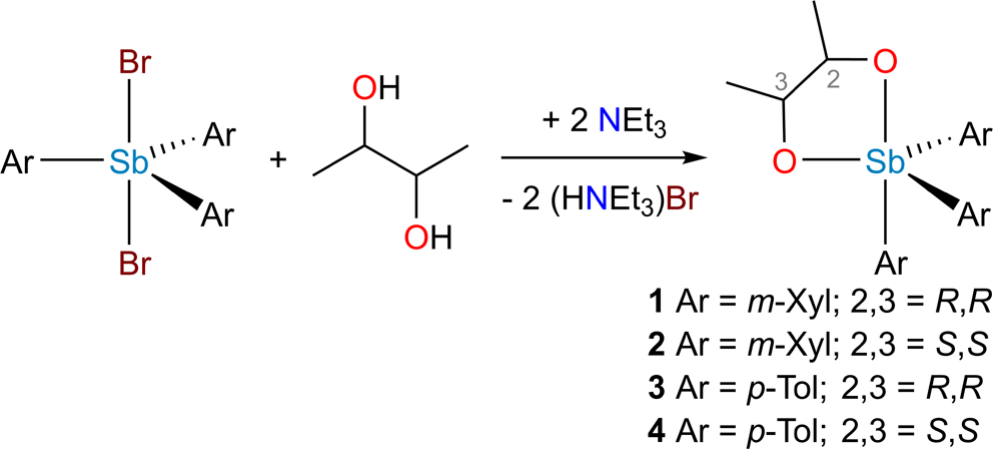
General Synthesis of **1**–**4**

We have previously described the synthesis of Sb(*p*-Tol)_3_L complexes, where L is a chelating diolate.^17^ These compounds were prepared to investigate polyalcohol chelation of Sb(V) centers while capitalizing on the favorable stability, solubility, and facile crystallization afforded by the triarylantimony(V) motif. For the present study, we used not only the previous tris(*para*-tolyl)antimony(V) scaffold but also expanded to include the tris(*meta*-xylyl)antimony(V) scaffold (Chart 1). The complexes were prepared by combining SbAr_3_Br_2_, the appropriate diol, and 2 equiv of Et_3_N (Scheme 1). The reaction with Sb(*m*-Xyl)_3_Br_2_ and 2*R*,3*R*-butanediol afforded a product that could be separated from the (HNEt_3_)Br byproduct via extraction with Et_2_O and recrystallized from MeCN. This procedure produced an analytically pure colorless crystalline solid, **1**. The ^1^H and ^13^C{^1^H} NMR spectra of **1** show a single set of *m*-Xyl signals and a single set of CH and CH_3_ resonances from the diolate. A rigid trigonal bipyramidal or square pyramidal structure would result in spectroscopically distinct sets of aryl and diolate signals. This behavior is analogous to that which we reported for related *p*-Tol complexes,^17^ and we invoke a similar degree of fluxionality to explain the spectra of **1**.

### Crystal Structure of **1**

**Figure 2:**
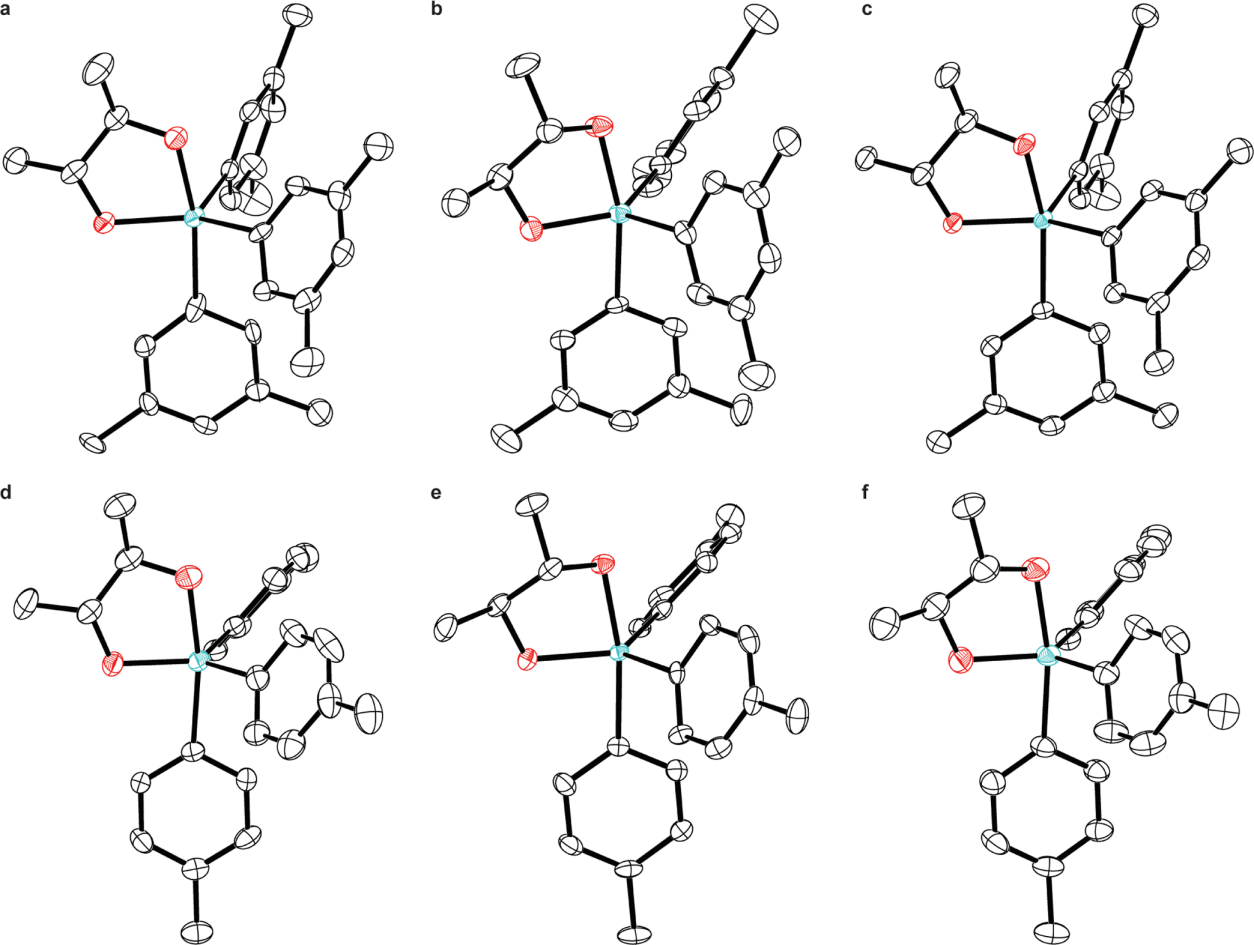
Thermal ellipsoid plots (50% probability) of (a) **1**, (b) **2**, (c) racemic compound **1**/**2**, (d) **3**, (e) **4**, and (f) racemic compound **3**/**4**. For crystals with *Z*′ = 2 (a, b, d, e), only one of the two crystallographically independent molecules is shown. H atoms omitted for clarity. Color code: Sb teal, O red, C black.

Single crystals of **1** grown by cooling an MeCN solution of the compound to −20 °C were analyzed by X-ray diffraction. It crystallized in space group *P*1 (Figure 2a and Table 1), with two full molecules in the asymmetric unit (*Z*′ = 2). The geometry about the Sb center is between trigonal bipyramidal and square pyramidal, and all of the bond lengths and angles align with those from our previously reported work on related *p*-Tol complexes.^17^ Inspection of the structure reveals that the two molecules in the asymmetric unit are approximately related by inversion. Allowing for inversion, the Sb(*m*-Xyl)_3_ fragments of the two molecules have an RMSD of 0.060 Å. Centroids calculated for pseudoinversion-related atoms within these fragments are virtually coincident, but as expected, the 2*R*,3*R*-butanediolate ligands do not obey this inversion symmetry.

**Table 1:** Crystallographic Data Collection and Refinement Parameters

	**1**	**2**	**1**/**2**	**3**	**4**	**3**/**4**
formula	C_28_H_35_O_2_Sb	C_28_H_35_O_2_Sb	C_28_H_35_O_2_Sb	C_25_H_29_O_2_Sb	C_25_H_29_O_2_Sb	C_25_H_29_O_2_Sb
FW	525.31	525.31	525.31	483.23	483.23	483.23
*T* (K)	105(7)	107(4)	108(4)	101(3)	107(2)	103(4)
λ (Å)	1.54184	1.54184	1.54184	1.54184	1.54184	1.54184
crystal system	triclinic	triclinic	triclinic	monoclinic	monoclinic	triclinic
space group	*P*1	*P*1	*P*1̅	*P*2_1_	*P*2_1_	*P*1̅
*a* (Å)	9.6092(2)	9.60900(10)	9.6369(2)	8.70780(10)	8.70850(10)	8.6690(3)
*b* (Å)	10.6217(3)	10.6249(2)	10.4594(2)	15.4287(2)	15.44630(10)	9.2367(3)
*c* (Å)	13.0406(2)	13.02660(10)	13.0831(3)	16.6758(2)	16.6628(2)	14.2286(5)
α (deg)	102.494(2)	102.4910(10)	102.195(2)			78.914(2)
β (deg)	91.5870(10)	91.6280(10)	92.245(2)	101.2980(10)	101.2130(10)	84.766(3)
γ (deg)	99.425(2)	99.3620(10)	99.022(2)			78.770(2)
volume (Å^3^)	1279.18(5)	1278.38(3)	1269.45(5)	2196.98(5)	2198.60(4)	1094.95(7)
*Z*	2	2	2	4	4	2
ρ_calc_ (mg m^–3^)	1.364	1.365	1.374	1.461	1.460	1.466
size (mm^3^)	0.07 × 0.05 × 0.03	0.16 × 0.07 × 0.06	0.31 × 0.27 × 0.21	0.19 × 0.11 × 0.07	0.27 × 0.23 × 0.08	0.10 × 0.08 × 0.05
θ range (deg)	3.479–68.238	3.483–68.246	3.466–68.222	2.702–68.239	3.938–68.251	3.170–68.251
total data	34,763	35,972	34,433	65,745	34,974	27,622
unique data	8873	8843	4650	8050	7888	4012
parameters	576	576	288	516	516	258
completeness (%)	100	99.9	99.9	100	99.8	99.9
*R*_int_ (%)	5.55	5.58	4.52	5.95	4.36	6.44
*R*_1_ (*I* > 2σ) (%)	3.03	3.38	2.51	2.63	3.54	3.42
*R*_1_ (all data) (%)	3.30	3.55	2.52	2.80	3.61	3.59
*w*R**_2_ (*I* > 2σ) (%)	7.42	8.80	6.47	7.36	9.39	8.03
*w*R**_2_ (all data) (%)	7.54	8.94	6.48	7.48	9.46	8.14
*S*	1.066	1.075	1.067	1.059	1.072	1.038
flack *x*	–0.001(15)	–0.008(14)		0.002(9)	0.014(8)	

It has been previously observed that in *Z*′ > 1 structures, symmetry-independent molecules are commonly related by pseudoinversion.^27−29^ This phenomenon arises, at least in part, because inversion symmetry is an effective way to achieve close packing.^30,31^ The effect of the pseudosymmetry in the structure of **1** was evident in reciprocal space as well; the heavy Sb atoms and our use of Cu Kα radiation resulted in significant anomalous dispersion. *R*_int_ calculated for crystallographic point group 1 was 5.5%, whereas that for crystallographic point group 1̅ was 6.5%.

### Synthesis and Crystal Structure of **2**

The analogous compound **2**, which bears a 2*S*,3*S*-butanediolate ligand, was readily prepared from Sb(*m*-Xyl)_3_Br_2_, NEt_3_, and 2*S*,3*S*-butanediol. As expected, the NMR spectra of **2** are identical to those of its enantiomer, **1**. Crystals of **2** suitable for X-ray diffraction could similarly be obtained by cooling an MeCN solution of the compound to −20 °C (Figure 2b). As noted above, the heavy Sb atoms ensured that there was sufficient anomalous dispersion to refine a Flack parameter with high precision, which was used to confirm the absolute structure.

### Synthesis and Crystal Structure of Racemic Compound **1/2**

We hypothesized that the crystal structure of the racemic compound formed from a mixture of **1** and **2** would be identical to that of either **1** or **2**, but with the pseudoinversion centers present as true crystallographic inversion centers. To test this proposal, a racemic solution in MeCN was prepared from isolated **1** and **2**. Cooling the solution to −20 °C resulted in the growth of diffraction-quality crystals. In contrast to the crystals of **1** and **2**, however, there was excellent agreement with Friedel’s Law (*R*_int_ = 4.5% for crystal point group 1̅). The structure was solved and refined without an issue in space group *P*1̅. The arrangement of the molecules is the same as in the structures of **1** and **2**, with the exception that the pseudoinversion centers are now true crystallographic inversion centers (Figure 2c).

The final refined unit cell parameters were nearly identical to those of **1** and **2** (Table 1), although the unit cell volume of the racemic compound is slightly smaller. The resulting increase in density for the racemic compound, as compared to either of the enantiomerically pure materials, aligns with Wallach’s rule.^32,33^ Although once thought to stem from a special innate affinity that one molecule has for an identical molecule of the opposite handedness, it is now appreciated that this increase in density (and stability) arises from an increase in close packing for the racemate. The slight violations of the pseudosymmetry in the structures of **1** and **2** prevent the inversion relation from achieving as close of a molecular packing as in the racemate, where the crystallographic inversion symmetry is rigorously obeyed.

### Extension to (2,3-Butanediolato)tris(*para*-tolyl)stiborane Compounds (**3** and **4**)

Having successfully predicted the behavior of the *meta*-xylyl-substituted species, we turned to the *para*-tolyl compounds. We had previously described the synthesis of **3** and the structure of a crystal obtained by slow evaporation of CDCl_3_ (space group *C*2).^17^ Using that same compound, we grew diffraction-quality crystals by cooling an MeCN solution to −20 °C. A new polymorph was obtained in space group *P*2_1_ (Figure 2d). As in the case of **1**, the molecule crystallized with *Z*′ = 2 and featured a pseudoinversion center between the crystallographically independent molecules in the asymmetric unit. The Sb(*p*-Tol)_3_ fragments from the two molecules featured an RMSD of only 0.104 Å after inversion. The *P*2_1_ and *C*2 polymorphs of **3** are compared in Figure S6.

The enantiomeric compound **4** was next prepared and crystallized. It was spectroscopically indistinct from **3** and diffraction-quality crystals could be grown from MeCN to afford an analogous *P*2_1_ structure (Figure 2e). The strong anomalous dispersion from the Sb atoms was used to confirm the absolute structure in each case. In both crystals, a pseudoinversion center was located at approximately *x* = 0.5 and *z* = 0.25. A true crystallographic inversion center at that location would result in a structure with space group *P*2_1_/*c*. We suspected that, as in the case of **1** and **2**, a combination of **3** and **4** would form a racemic compound **3**/**4** with the same unit cell as both **3** and **4** (perhaps marginally smaller) but with the pseudosymmetries being converted to true crystallographic symmetries. As a consequence, the space group would be *P*2_1_/*c*. A racemic solution of **3** and **4** was prepared and cooled to −20 °C, which yielded diffraction-quality, colorless crystals. Unexpectedly, the diffraction pattern did not have 2/*m* symmetry and was best indexed using a triclinic unit cell. No particular relationships were present between the elements of the Niggli matrix to indicate that the crystal had the metric symmetry required by the monoclinic crystal system.^34,35^ The structure could be solved in *P*1̅ with *Z*′ = 1 and refined without complication (Figure 2f).

**Figure 3:**
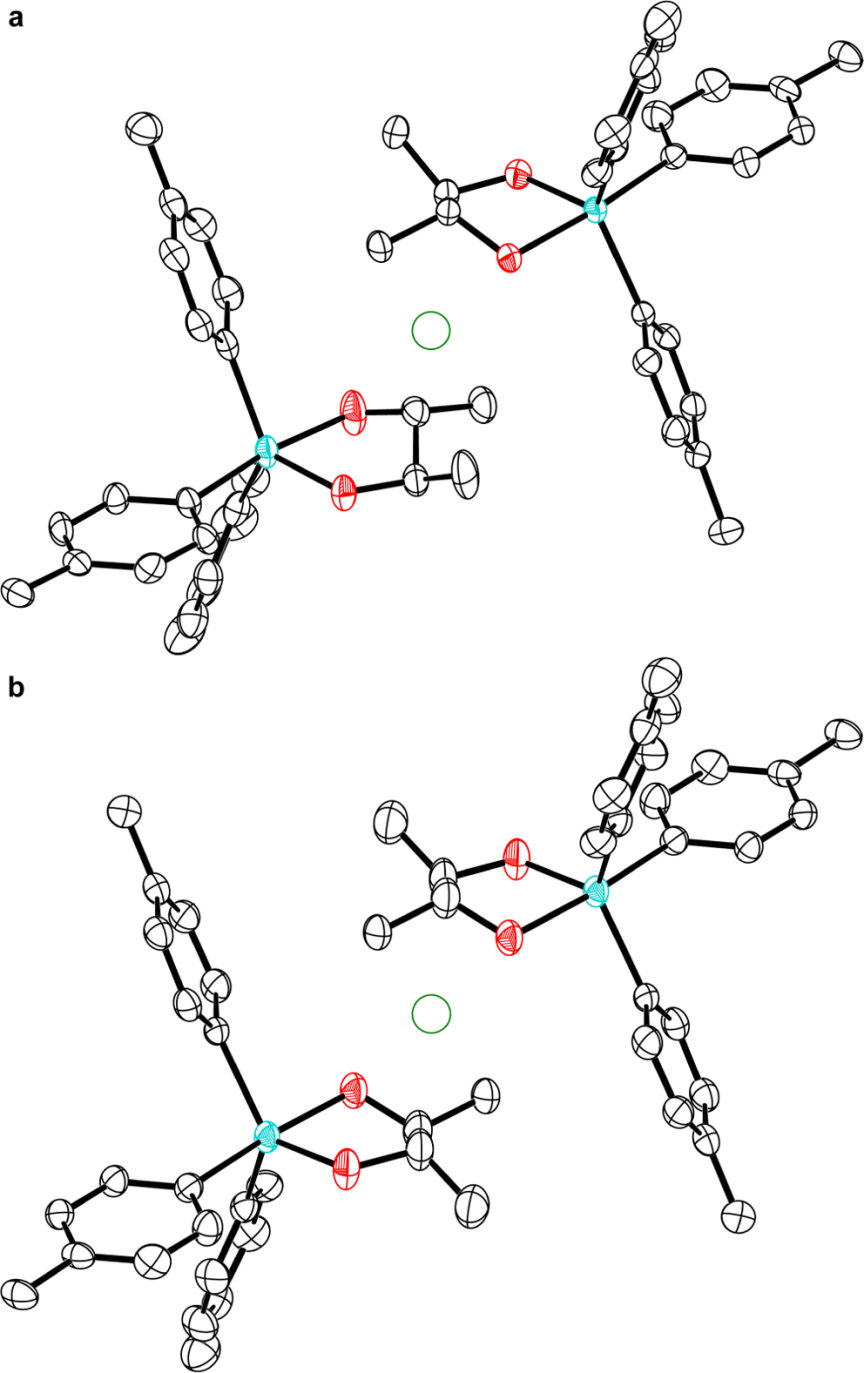
(a) Asymmetric unit of **3** and (b) unit cell of racemic compound **3**/**4**. Anisotropic displacement ellipsoids are drawn at the 50% probability level, and H atoms are omitted for clarity. The (pseudo)inversion centers are shown as green circles. Color code: Sb teal, O red, C black.

As in the case of the xylyl-substituted species described above, the density of the crystal of racemic compound **3**/**4** was slightly higher than that of either of the individual enantiomerically pure species (Table 1). The pseudoinversion centers that had related the contents of the asymmetric units of the crystal structures of **3** and **4** were now true inversion centers in the *P*1̅ structure (Figure 3). Although this pairwise (pseudo)symmetry is the same across the structures, the overall crystal structure of the racemic compound did not maintain the framework of the enantiomerically pure species, which would have afforded a structure in space group *P*2_1_/*c*.

**Figure 4:**
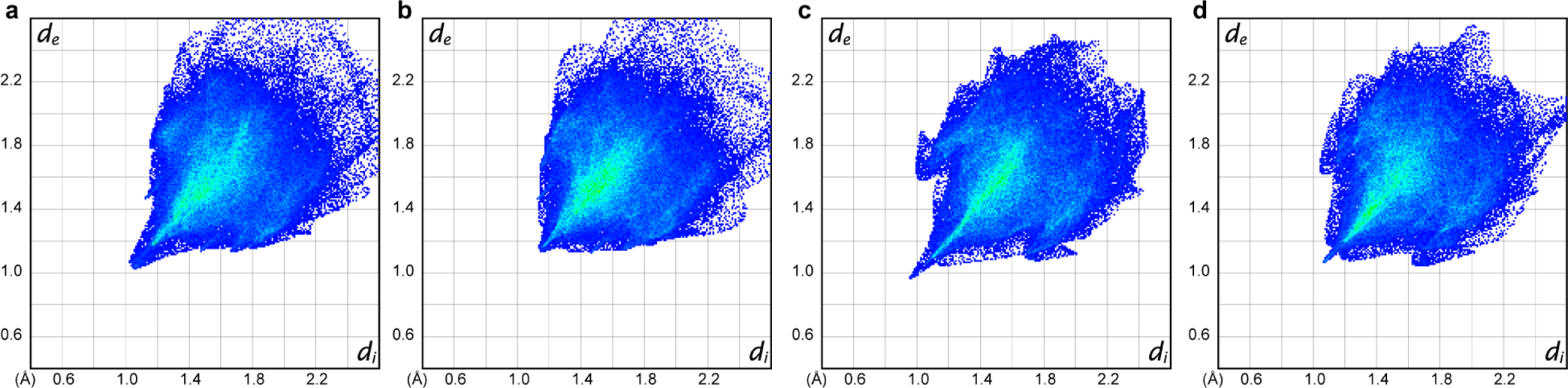
Two-dimensional fingerprint plots showing the distance from the Hirshfeld surface of the molecule to the nearest atom interior to the surface (*d*_i_) and the distance from the Hirshfeld surface of the molecule to the nearest atom exterior to the surface (*d*_e_) for (a) racemic compound **1**/**2**, (b) **2**, (c) racemic compound **3**/**4**, and (d) **3**.

Given the similarity between the individual molecules from the crystal structures of **3**, **4**, and racemic compound **3**/**4**, as well as the similarity between the arrangement of pairwise (pseudo)inversion-related molecules, we sought to probe the structures more generally. One means of capturing the complex intermolecular interactions present in a crystalline solid is to map the Hirshfeld surface of a molecule and then to calculate the distances from that surface to atoms inside (*d*_i_) and outside (*d*_e_) the surface.^23,24^ Two-dimensional plots in which bins are populated with the number of atoms that have a given combination of *d*_i_ and *d*_e_ values have become popular tools for fingerprinting intermolecular interactions in crystals.^25^ We first performed the analysis of the xylyl-substituted species using the single molecule in the asymmetric unit of **1**/**2** (Figure 4a) and one of the crystallographically independent molecules in the asymmetric unit of **1** (Figure 4b). Although there are subtle differences between the two, the overall shapes of the plots are largely the same. These fingerprint plots capture information about the local structure of the crystal in the vicinity of a molecule, and the similarities between the plots in Figure 4a,b are consistent with the approximate congruence of the crystal structures.

In the analysis of **3**/**4** (Figure 4c) and **3** (Figure 4d), we again observed similarities between the general structure and the fine features of the two fingerprint plots. Although the racemic compound **3**/**4** had crystallized in an altogether different crystal system and space group, this result suggests there are overall similarities in the local packing structures of the two crystals.

**Figure 5:**
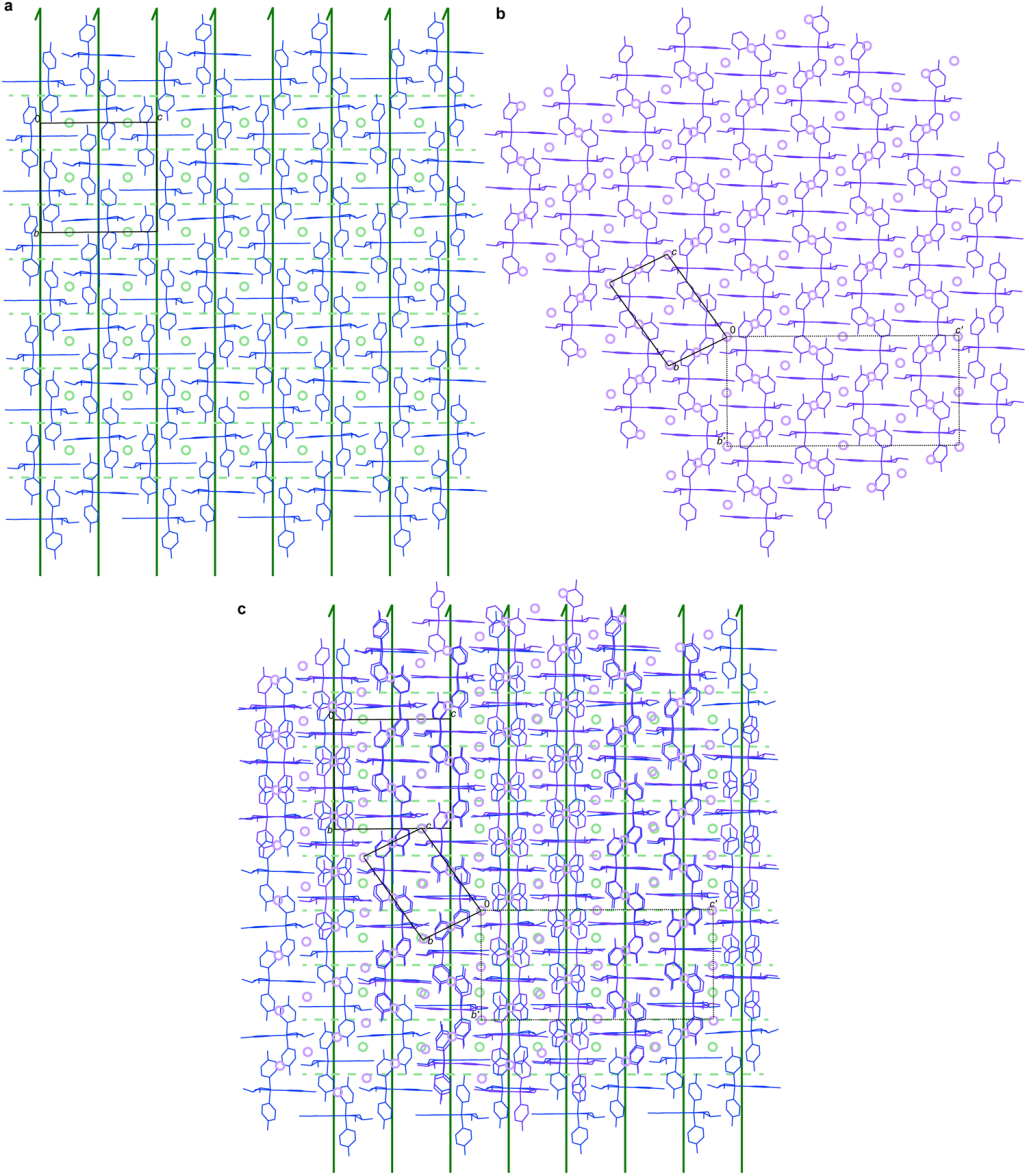
(a) Packing diagram of **3** viewed along *a*. Molecules are shown as blue sticks, the unit cell as black lines with origin and axes labeled, 2_1_ screw axes as dark green arrows, pseudoinversion centers as pale green circles, and pseudo-*c* glide planes as pale green dashed lines. (b) Packing diagram of the racemic compound **3**/**4** viewed along *a*. Molecules are shown as purple sticks, the unit cell as black lines with origin and axes labeled, the transformed unit cell as dotted black lines with origin and axes labeled, and the inversion centers as lavender circles. (c) An overlap of the structures of **3** (blue) and racemic compound **3**/**4** (purple) both maintained in the orientations depicted in (a) and (b). All symmetry elements, pseudosymmetry elements, and unit cells are depicted as described above.

The unit cell of **3**/**4** can be transformed into one that approximates the *P*2_1_ unit cell of **3** using the matrix (−1 0 0 | 0 –1 1 | 0 1.5 0.5). This transformed cell does not, however, capture the translational symmetry of the crystal in the *c*′ direction. A transformed cell in which the longest axis (*c*′) is doubled affords a translationally valid unit cell (dotted lines in Figure 5b) and can be obtained by transforming the original triclinic unit cell with the matrix (−1 0 0 | 0 –1 1 | 0 3 1).

The overlay of the structures (Figure 5c) highlights the link between them. Along the *c* axis of the *P*2_1_ unit cell of **3**, there are regularly spaced pseudoinversion (**3**) or inversion (**3**/**4**) centers. In an alternating fashion, every second pseudoinversion center of **3** along *c* coincides approximately with an inversion center of **3**/**4**. The pseudoinversion centers of **3** that do not overlap with the inversion centers of **3**/**4** are arranged in columns parallel to *b* at *z* = 0.75 (Figure 5). Inspection of the overlap highlights that the structure of **3**/**4** also has inversion centers arranged in columns parallel to *b*′, but that they are out of register with the pseudoinversion centers of **3** by 0.25*b′*.

The structure of **3** features columns of molecules aligned parallel to the *b* axis that are related by 2_1_ screw rotations. In the structure of **3**/**4**, there are columns of molecules similarly arrayed parallel to *b*′ but they are related locally by pseudo-2_1_ screw symmetry. The columns of molecules at *z* = 0 and 0.5 overlap approximately, but the displacement of the inversion centers at *z* = 0.75 results in a lack of overlap between the molecules at *z* = 1 and 1.5; the columns from **3** and **3**/**4** are out of register in the *y* direction by 0.5*b*. At *z* = 2, the structures overlap again.

## Discussion

The xylyl-substituted compounds **1** and **2** both crystallized in space group *P*1, consistent with their enantiomerically pure nature. This space group has frequently attracted attention because of how often structures are incorrectly assigned to it,^27,35−39^ although the situation has improved over time.^40^ Inversion symmetry has long been recognized as one of the most favored crystal-packing motifs,^41^ and in a survey of database-deposited crystal structures, it was estimated that about one-third of structures in which chiral molecules crystallized in space group *P*1 featured approximate centrosymmetry.^27^ Further analyses have highlighted that crystals of homochiral substances, which cannot form in centrosymmetric space groups, often arrange in a way that mimics inversion symmetry.^15^ Moreover, it has been noted that when a molecule has only two stereocenters that are adjacent, simultaneous reversal of the handedness of both stereocenters frequently has a relatively minor impact on the shape of the overall molecular envelope.^29^ This effect on the overall molecular shape is particularly minimal when each stereocenter has an H atom substituent, as is the case with the 2,3-butanediolate complexes studied here. Because the molecular envelopes of **1** and **2** are highly similar, a collection of either only **1** (or **2**) molecules can pack in a fashion analogous to the racemate. The fingerprint plots in Figure 4 highlight the similarities between these packings. This similarity in packing led to our proposal that the racemic compound **1**/**2** would crystallize in a manner isomorphous to **1** and **2** but in space group *P*1̅. This was indeed the case, highlighting the potential for such observations to be used in crystal design.

The case of **3** and **4** differed. Although the homochiral species crystallized in *P*2_1_ with pseudoinversion centers positioned such that the structure approximates *P*2_1_/*c*, racemic compound **3**/**4** did not crystallize in space group #14. Instead, it underwent an apparently dramatic change from monoclinic to triclinic, an alteration in the unit cell basis vectors, a halving of the unit cell volume, and a change in the space group from *P*2_1_ to *P*1̅. An initial analysis revealed, however, that many of the features of the local molecular packing are maintained across both structures. Moreover, an analysis of the extended structures reveals that they are indeed quite closely related. The racemic compound did not simply maintain the same columnar packing as the enantiomerically pure compound with a change of pseudoinversion centers for true inversion centers. Instead, half of the columns of molecules are shifted by half a unit cell length along their column axis. Although this shift breaks many of the long-range symmetries that would be present if a simple pseudoinversion-to-inversion change had occurred, it highlights that many elements of symmetry and pseudosymmetry are preserved.

A possible consequence of the preservation of the overall packing between **1** and **1**/**2** is that **1** and **2** may be able to form solid solutions: isostructural phases that contain variable proportions of the two enantiomers.^42,43^ It may be the case that solid solutions are particularly favored by otherwise achiral molecules that feature two adjacent stereocenters that each bear an H atom and feature these H atoms in a trans disposition.^29^ Situations such as those of **3** and **3**/**4** will provide an interesting point of comparison because there is a relationship between their three-dimensional structures, but they are not isostructural. We look forward to investigating the ability of these compounds to form solid solutions.

Finally, it is noteworthy that the presently discussed structures are held together exclusively by van der Waals interactions. Lower-symmetry homochiral crystals that feature pseudoinversion centers but also have stronger, directed intermolecular interactions could allow for a more reliable conversion of a lower-symmetry structure with global pseudosymmetry into the corresponding higher-symmetry crystal structure by growing crystals from racemic solutions.

## Conclusions

In this work, we explored complexes of *trans*-2,3-butanediolates with two different triarylantimony(V) scaffolds, where aryl = *meta*-xylyl and *para*-tolyl. For the *meta*-xylyl species, the 2*R*,3*R*-butanediolate and 2*S*,3*S*-butanediolate complexes **1** and **2** formed isostructural crystals in space group *P*1 with *Z*′ = 2. A pseudoinversion center relating the molecules in the asymmetric unit caused the structure to approximate the space group *P*1̅. We hypothesized that the racemate would preserve the intermolecular packing interactions but that the pseudoinversion centers would become true crystallographic inversion centers. The racemic compound **1**/**2** did indeed form isostructural crystals in the space group *P*1̅. For the *para*-tolyl species, the 2*R*,3*R*-butanediolate and 2*S*,3*S*-butanediolate complexes **3** and **4** formed isostructural crystals in space group *P*2_1_ (*Z*′ = 2) and a pseudoinversion center relates the molecules in the asymmetric unit. The homochiral structures approximated space group *P*2_1_/*c*, but racemic compound **3**/**4** crystallized in triclinic space group *P*1̅. An analysis of the tolyl-substituted structures reveals that, in addition to many aspects of local intermolecular interactions being maintained, there are key large-scale similarities between the three-dimensional structures of racemic compound **3**/**4** and the constituent homochiral species. It must also be stressed that although **3**/**4***did* not yield a *P*2_1_/*c* structure when crystallized from MeCN under conditions identical to those that yielded *P*2_1_ structures of **3** and **4** approximating *P*2_1_/*c*, we have not demonstrated that a *P*2_1_/*c* structure of **3**/**4***can* not form. Further variation of crystallization conditions may indeed afford the hypothesized *P*2_1_/*c* structure.

## Supporting Information Available

NMR data; thermal ellipsoid plots; crystallographic tables (PDF)
