# Supplementary material for: Exploring the Use of Pseudosymmetry in the Design of Higher-Symmetry Crystals of Racemic Compounds
Source: Cryst Growth Des. 2024 Nov 25;24(24):10247–55. doi: 10.1021/acs.cgd.4c01240 (PMC11660144; doi:10.1021/acs.cgd.4c01240)
Supplement: Supplementary file 1 — cg4c01240_si_001.pdf [file cg4c01240_si_001.pdf]

Supporting Information for:

Exploring the Use of Pseudosymmetry in the Design of Higher-Symmetry Crystals of Racemic Compounds

Brent Lindquist-Kleissler, [a] *Viky Villanueva,* [a] *Addis Getahun,* [a] *and Timothy C. Johnstone** [a]

[a] Department of Chemistry and Biochemistry, University of California Santa Cruz, Santa Cruz, California 95064, United States.

Correspondence: johnstone@ucsc.edu

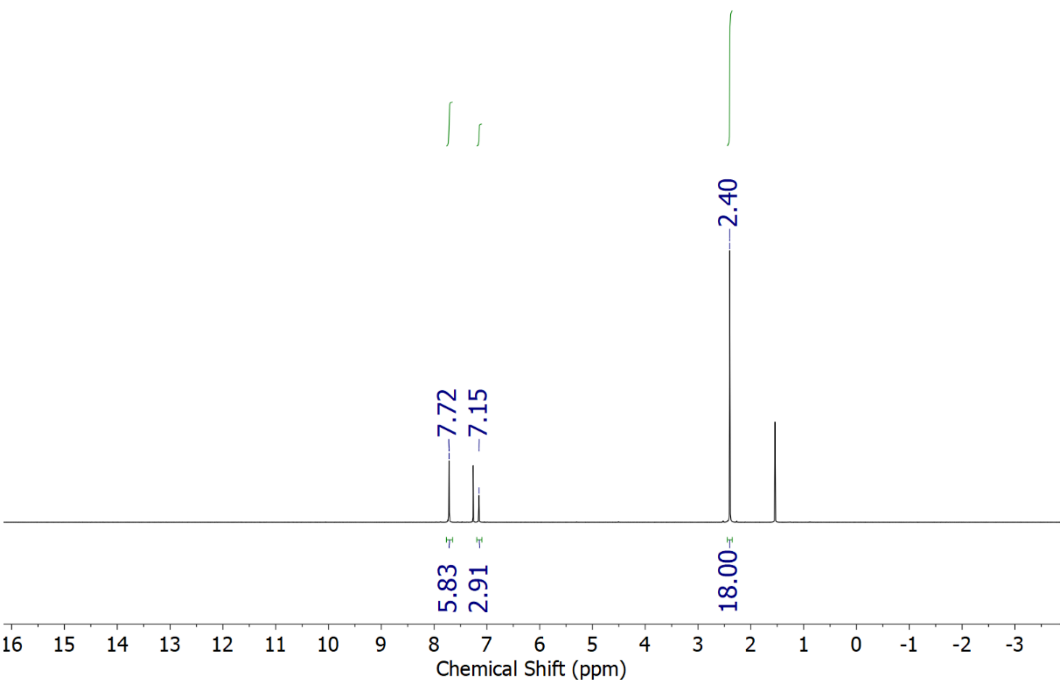

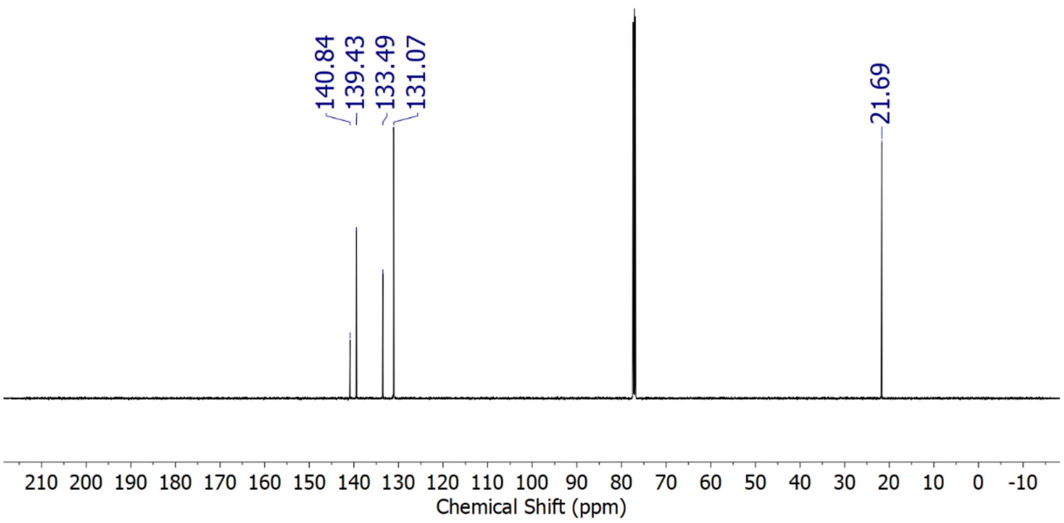

Figure S1. *Top*: ^{1}H NMR (500 MHz, $CDCl_3$) spectrum of Sb(*m*-Xyl)$_3$Br$_2$. *Bottom*: ^{13}C{^{1}H} NMR (126 MHz, $CDCl_3$) spectrum of Sb(*m*-Xyl)$_3$Br$_2$.

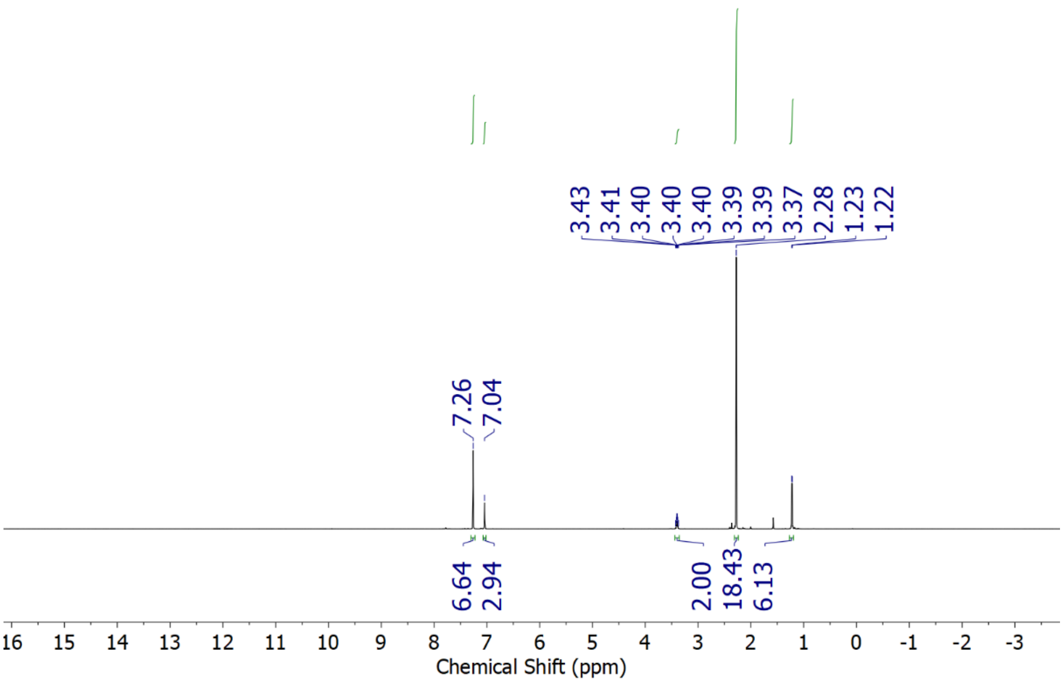

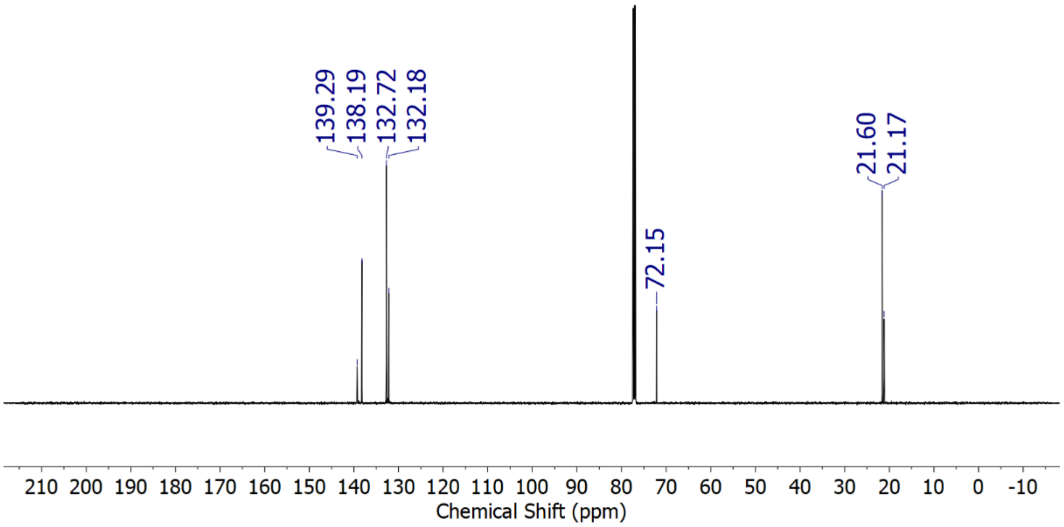

Figure S2. *Top*: ^{1}H NMR (500 MHz, $CDCl_3$) spectrum of **1**. *Bottom*: $^{13}C\{^1H\}$ NMR (126 MHz, $CDCl_3$) spectrum of **1.**

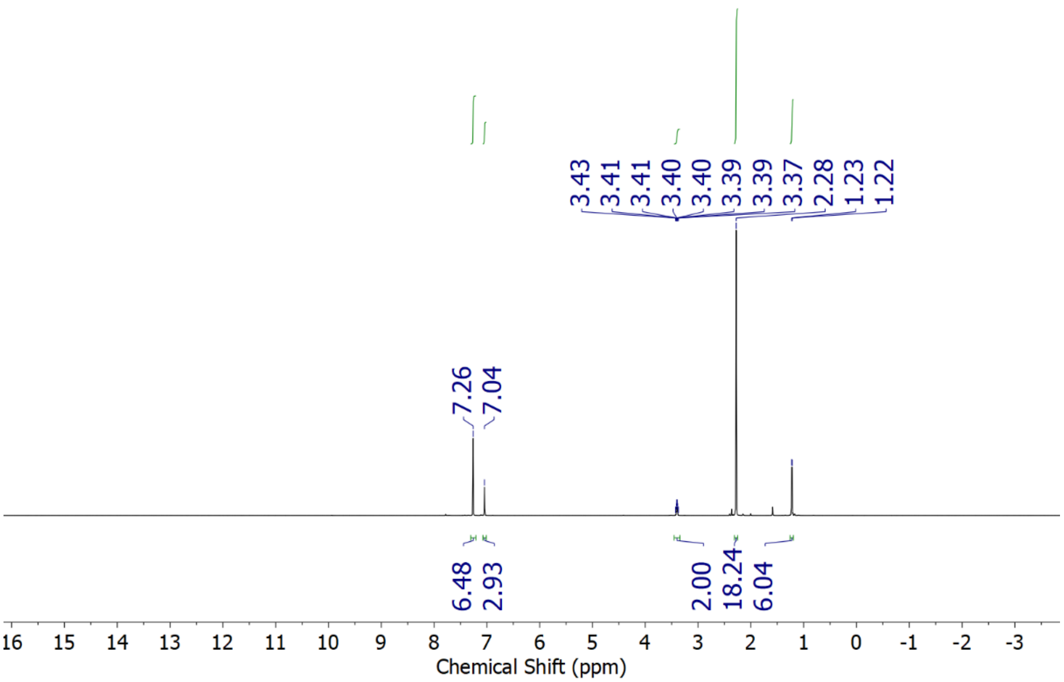

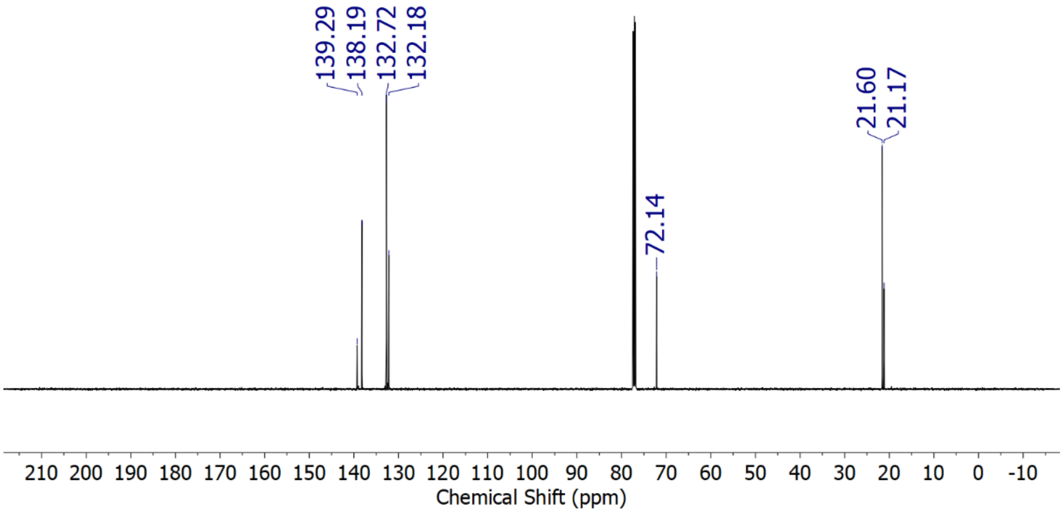

Figure S3. *Top*: ^{1}H NMR (500 MHz, $CDCl_3$) spectrum of **2**. *Bottom*: $^{13}C\{^{1}H\}$ NMR (126 MHz, $CDCl_3$) spectrum of **2**.

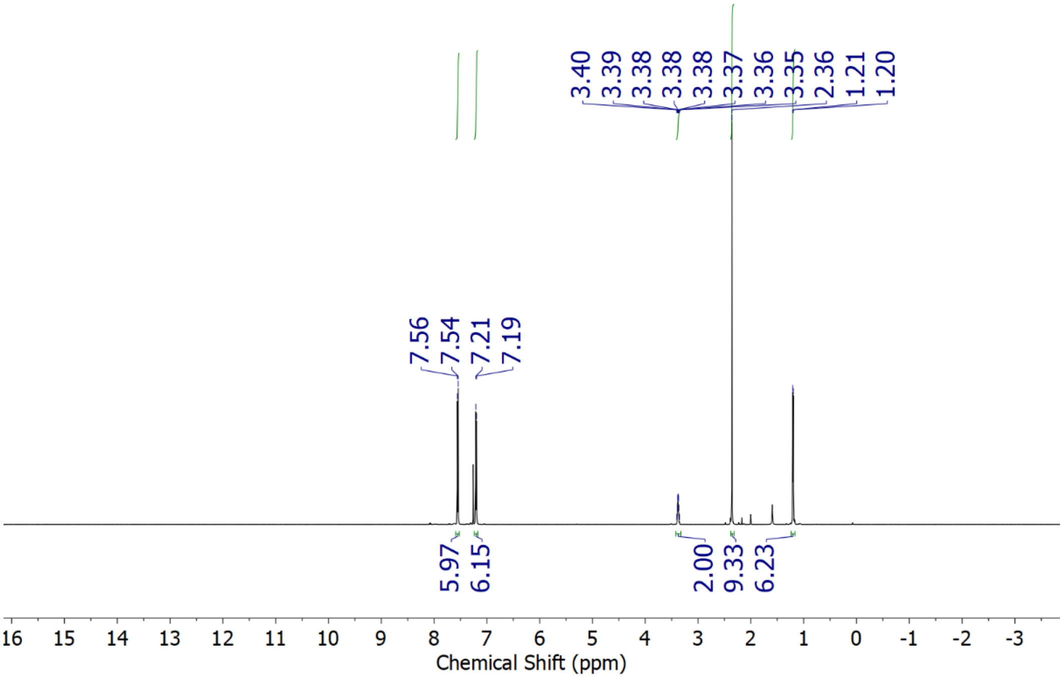

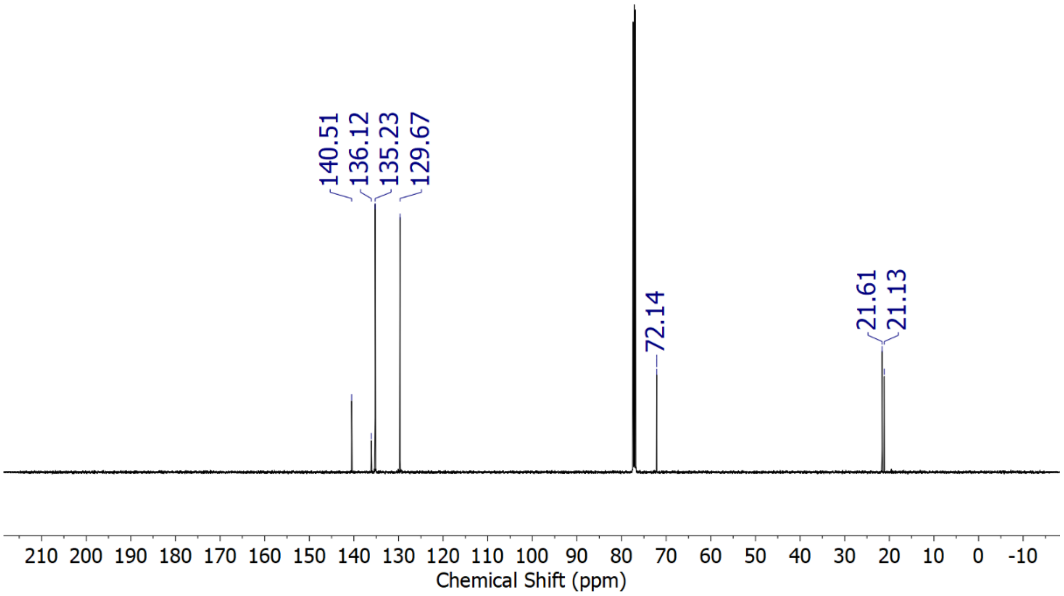

Figure S4. *Top*: ^{1}H NMR (500 MHz, $CDCl_3$) spectrum of **4**. *Bottom*: $^{13}C\{^{1}H\}$ NMR (126 MHz, $CDCl_3$) spectrum of **4.**

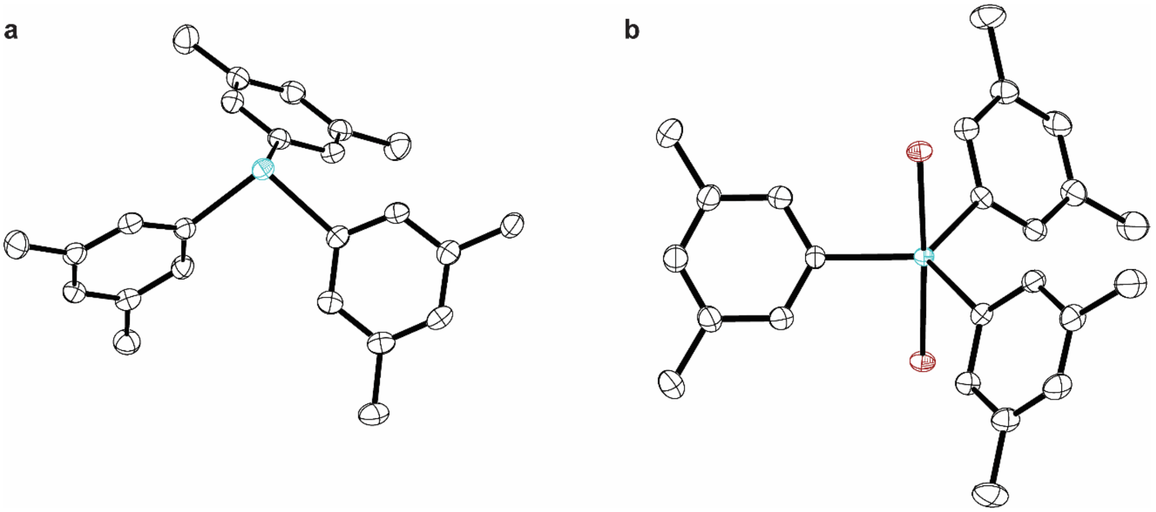

Figure S5. Thermal ellipsoid plots (50% probability) of (a) Sb(*m*-Xyl)$_3$ and (b) Sb(*m*-Xyl)$_3$Br$_2$. H atoms omitted for clarity. Color code: Sb teal, C black, Br maroon.

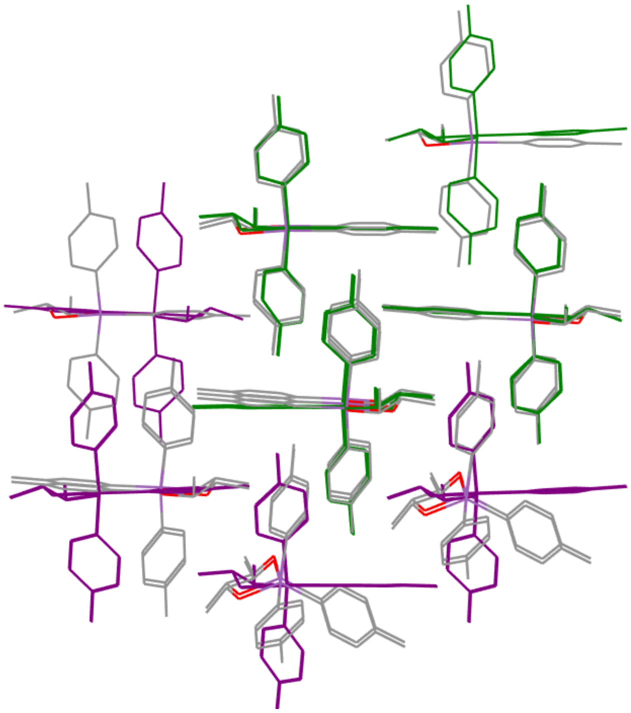

Figure S6. Overlay of one unit cell of **3** crystallized from chloroform (element-colored sticks, C grey; *C*2 structure from previous report [*Dalton Trans.* **2023,** *52*, 9229-9237]) and an equivalent number of molecules from the structure obtained when **3** was crystallized from acetonitrile (green or purple sticks; *P*2$_1$ structure from present work). Two unit cells' worth of molecules of the *P*2$_1$ structure were needed. The green molecules from the *P*2$_1$ structure overlap with those of the *C*2 structure, the purple molecules do not.

Table S1. Crystallographic Data and Refinement Parameters

	**Sb(*m*-Xyl)$_3$**	**Sb(*m*-Xyl)$_3$Br$_2$**
Formula	$C_{24}H_{27}Sb$	$C_{24}H_{27}Br_2Sb$
FW	437.20	597.02
T (K)	103(4)	108(3)
λ (Å)	1.54184	1.54184
Crystal system	Monoclinic	Orthorhombic
Space group	$P2_1/c$	*Pbcn*
a (Å)	8.60420(10)	16.1490(2)
b (Å)	12.6653(2)	10.3012(2)
c (Å)	18.8060(2)	14.0848(2)
β (°)	91.5170(10)	
Volume (Å^3)	2048.66(5)	2343.06(6)
Z	4	4
ρ_{calc} (Mg m^{-3})	1.418	1.692
Size (mm^3)	0.10×0.07×0.05	0.16×0.07×0.06
θ range (°)	4.209–68.245	5.408–68.216
Total data	31338	27124
Unique data	3752	2142
Parameters	232	127
Completeness (%)	100	100
R_{int} (%)	4.29	5.25
R_1 (I > 2σ) (%)	2.27	1.93
R_1 (all data) (%)	2.48	2.04
wR_2 (I > 2σ) (%)	5.99	5.17
wR_2 (all data) (%)	6.11	5.25
S	1.054	1.036
